# Muscle mass, strength and functional outcomes in critically ill patients after cardiothoracic surgery: does neuromuscular electrical stimulation help? The Catastim 2 randomized controlled trial

**DOI:** 10.1186/s13054-016-1199-3

**Published:** 2016-01-29

**Authors:** Arabella Fischer, Matthias Spiegl, Klaus Altmann, Andreas Winkler, Anna Salamon, Michael Themessl-Huber, Mohamed Mouhieddine, Eva Maria Strasser, Arno Schiferer, Tatjana Paternostro-Sluga, Michael Hiesmayr

**Affiliations:** 1 Division of Cardiothoracic and Vascular Anesthesia and Intensive Care Medicine, Department of Anesthesia, General Intensive Care and Pain Control, Medical University of Vienna, Währingergürtel 18-20, 1090 Vienna, Austria; 2 Kantonsspital Sankt Gallen, Rorschacherstrasse 95, 9007 St. Gallen, Switzerland; 3 Krankenhaus der Barmherzigen Schwestern Ried, Schlossberg 1, 4910 Ried im Innkreis, Austria; 4 University Hospital Landeskrankenhaus Salzburg, Müllner Hauptstraße 48, 5020 Salzburg, Austria; 5 Medical University of Vienna, Spitalgasse 23, 1090 Vienna, Austria; 6 Center for Medical Statistics, Informatics and Intelligent Systems, Medical University of Vienna, Spitalgasse 23, 1090 Vienna, Austria; 7 Institute for Physical Medicine and Rehabilitation, Kaiser-Franz-Josef-Spital Wien, Kundratstrasse 3, 1100 Vienna, Austria; 8 Institute for Physical Medicine and Rehabilitation, Donauspital Wien, Langobardenstraße 122, 1220 Vienna, Austria

**Keywords:** Electrical stimulation therapy, ICU-acquired weakness, Muscular atrophy, Ultrasonography, Muscle layer thickness, Edema, Muscle strength, Critically ill, Cardiothoracic surgery, Functional outcomes

## Abstract

**Background:**

The effects of neuromuscular electrical stimulation (NMES) in critically ill patients after cardiothoracic surgery are unknown. The objectives were to investigate whether NMES prevents loss of muscle layer thickness (MLT) and strength and to observe the time variation of MLT and strength from preoperative day to hospital discharge.

**Methods:**

In this randomized controlled trial, 54 critically ill patients were randomized into four strata based on the SAPS II score. Patients were blinded to the intervention. In the intervention group, quadriceps muscles were electrically stimulated bilaterally from the first postoperative day until ICU discharge for a maximum of 14 days. In the control group, the electrodes were applied, but no electricity was delivered. The primary outcomes were MLT measured by ultrasonography and muscle strength evaluated with the Medical Research Council (MRC) scale. The secondary functional outcomes were average mobility level, FIM score, Timed Up and Go Test and SF-12 health survey. Additional variables of interest were grip strength and the relation between fluid balance and MLT. Linear mixed models were used to assess the effect of NMES on MLT, MRC score and grip strength.

**Results:**

NMES had no significant effect on MLT. Patients in the NMES group regained muscle strength 4.5 times faster than patients in the control group. During the first three postoperative days, there was a positive correlation between change in MLT and cumulative fluid balance (*r* = 0.43, *P* = 0.01). At hospital discharge, all patients regained preoperative levels of muscle strength, but not of MLT. Patients did not regain their preoperative levels of average mobility (*P* = 0.04) and FIM score (*P* = 0.02) at hospital discharge, independent of group allocation.

**Conclusions:**

NMES had no effect on MLT, but was associated with a higher rate in regaining muscle strength during the ICU stay. Regression of intramuscular edema during the ICU stay interfered with measurement of changes in MLT. At hospital discharge patients had regained preoperative levels of muscle strength, but still showed residual functional disability and decreased MLT compared to pre-ICU levels in both groups.

**Trial registration:**

Clinicaltrials.gov identifier NCT02391103. Registered on 7 March 2015.

**Electronic supplementary material:**

The online version of this article (doi:10.1186/s13054-016-1199-3) contains supplementary material, which is available to authorized users.

## Background

Intensive care unit-acquired weakness (ICUAW) affects 24–77 % of patients with an ICU stay longer than 1 week [1]. The main risk factors for ICUAW development include sepsis, catecholamine administration, hyperglycemia, ICU length of stay and immobility [1]. ICUAW is associated with symptomatic aspirations [2] and increased hospital mortality [1].

At 5 years after ICU discharge, the 6-minute walking distance (6MWD) and the quality of life assessed by the 36-Item Short Form (SF-36) questionnaire are still decreased [3]. Activities in daily living and cognitive function are impaired even up to 8 years after ICU discharge [4]. In order to prevent these tremendous consequences of ICUAW, early diagnosis and treatment may be decisive. In nonsedated patients, ICUAW is diagnosed when Medical Research Council (MRC) sum score is below 48 out of 60 points or when mean MRC score is below 4 out of 5 points in all testable muscle groups [5]. In sedated patients, muscle ultrasonography may be a diagnostic tool to detect muscle wasting [6, 7]. It is unclear whether muscular ultrasonography is influenced by edema [6–13]. Among different treatment modalities of ICUAW [14, 15], neuromuscular electrical stimulation (NMES) is a feasible therapy for neuromuscular activation in sedated patients. Yet, the effects of NMES on muscle mass and strength are still unclear in critically ill patients: randomized controlled trials either showed no effect [11, 16, 17] or (partially) beneficial effects [9, 17–22]. Moreover, most prior NMES studies did not assess any functional outcomes at hospital discharge to assess the potential effect of NMES on the patient's functional status after the ICU stay [9, 11, 17, 19–21]. NMES can be safely applied in critically ill patients with external pacemakers after cardiothoracic surgery [23, 24].

To date, no randomized controlled trial has evaluated the effects of NMES on muscle mass and strength in a cardiothoracic surgery patient population. Given that cardiovascular disease is the leading cause of death worldwide [25], more attention needs to be addressed to the rehabilitation of critically ill patients after cardiac surgery. Neither have trials assessed muscle mass, strength and functional status altogether prior to critical illness, which is necessary for evaluating change in muscle mass and strength in relation to preoperative values.

Therefore the first objective was to investigate whether early NMES is effective in preventing loss of MLT and strength in critically ill patients after cardiothoracic surgery. The second objective was to observe the time variation of muscle layer thickness (MLT) and muscle strength from preoperative day to hospital discharge. To adjust for possible confounders for measurement of MLT, daily fluid balance was assessed during the ICU stay.

## Methods

### Study design and population

The prospective, randomized, controlled, single-blind trial Catastim 2 was carried out at the Division of Cardiothoracic and Vascular Anesthesia and Intensive Care Medicine of the General Hospital affiliated to the Medical University of Vienna from May 2011 to July 2012 (clinicaltrials.gov identifier: NCT02391103). Patients were blinded to the NMES/sham stimulation. Nonblinded assessors performed the ultrasound scans; however, the evaluators measuring MLT were blinded to patients’ group assignment. The assessors of muscle strength and the secondary outcomes were not blinded to patients’ group assignment. Ethical approval was obtained from the ethics committee of the Medical University of Vienna in 2010 (number 1072/2010). Patients were included if they underwent cardiothoracic surgery and were anticipated to stay in the ICU for at least 48 hours. Patients were excluded if they were younger than 18 years, had a body mass index of more than 40 kg/m^2^, had metal implants or skin lesions in the stimulation area, had neuromuscular diseases, had an implanted ventricular assist device or intra-aortic balloon pump. After submission of a protocol amendment in June 2011, a twofold study setting was defined in order to recruit patients not only before, but also after cardiothoracic surgery. In cohort A, patients were recruited before surgery. In cohort B, patients were recruited after surgery. Recruitment of patients after surgery primarily allowed inclusion of patients after urgent nonelective surgeries such as heart transplantations, who could not be seen preoperatively. Written informed consent was obtained from patients in cohort A. Patients in cohort B were included in the trial while sedated to be later informed of their participation. The inclusion of sedated patients in cohort B without providing written informed consent was approved by the ethics committee of the Medical University of Vienna.

### Randomization

For all patients, randomization was performed on postoperative day 1. Patients in cohort A and B were separately randomized using a web-based randomizer for clinical trials [26]. In the previous Catastim 1 trial taking place in the same ICU [24], the NMES and control groups had slightly different Simplified Acute Physiology Score II (SAPS II) scores. Hence, in the Catastim 2 trial, randomization was stratified by the SAPS II score on the first postoperative day to ensure balance of the NMES and control groups with respect to disease severity. The four strata were deduced from the quartiles of SAPS II scores in a retrospective analysis of 2708 patients of the same ICU.

### Intervention

In the intervention group, all parts of the quadriceps muscle (rectus femoris muscle, vastus intermedius muscle, vastus lateralis muscle, vastus medialis muscle) of both thighs were electrically stimulated. The muscles were stimulated twice a day (2 × 30 minutes of NMES with an interval of at least 30 minutes between both sessions [24]) 7 days a week during the entire ICU stay but no longer than 14 days, starting on postoperative day 1. Highest tolerable intensity just below the pain threshold was applied. A visible and palpable contraction was the goal to achieve. The Compex 3 Professional (CefarCompex Medical AB, Freiburg, Germany) stimulator delivered biphasic rectangular pulses of 0.4 ms pulse duration at 66 Hz [19]. The duty cycle was 3.5 s on and 4.5 s off, ramping up and down were both set at 0.5 s [24]. Dual Snap (5 × 10 cm and 5 × 5 cm) electrodes (CefarCompex Medical AB) were used. In the control group, the electrodes were applied, connected to the stimulator, but no electricity was delivered. Patients were blinded to the intervention: All patients were told they would get NMES, whether or not electricity was delivered.

### Outcomes

#### Muscle layer thickness (MLT)

**Fig. 1:**
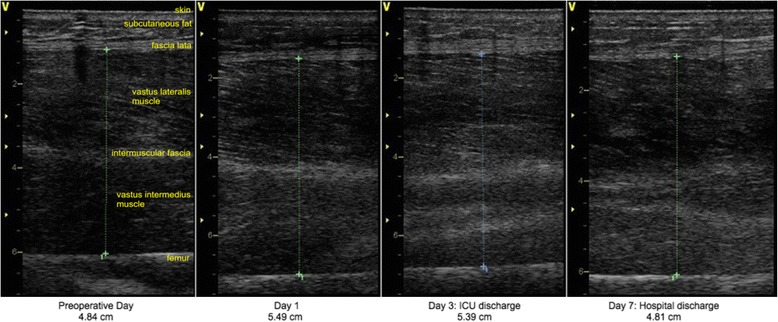
Ultrasound scans of the left thigh at the lateral measuring point in the sagittal plane (Patient no. 104, control group)

For all patients, MLT of the quadriceps muscle of both thighs using two-dimensional B-mode ultrasound (Vivid i, General Electric Healthcare, Little Chalfont, UK) was measured on postoperative day 1, every other day until ICU discharge and at hospital discharge. Patients in cohort A had an additional assessment of MLT before surgery. In cohort A, the preoperative assessment took place on the preoperative day in most patients (*n* = 23). Due to postponement of surgery, the preoperative assessment was between 2 and 8 days before surgery in six patients. In previous studies [7, 27, 28] the cross-sectional area of the rectus femoris muscle was measured. However, the rectus femoris muscle only constitutes approximately 10 % of the total quadriceps cross-sectional area [29]. Even though there is no data about differing wasting rates of the quadriceps muscle components in critically ill patients, hypertrophy may occur at different rates in the quadriceps muscle components in healthy young males during training [30]. Thus we measured not a cross-sectional area of a single muscle, but the muscle layer thickness of all parts of the quadriceps muscle. During the ICU stay, edema may enlarge the cross-sectional area of the muscle. Furthermore the ultrasound transducer is only 3.8 cm wide. Thus it is impossible to measure all parts of the quadriceps muscle at only one measuring point by ultrasonography. In order to account for all parts of the quadriceps muscle in the most reliable way, we used three measuring points per leg in two orthogonal planes to increase precision. Twelve measurements per patient per observation day were averaged to account for all parts of the quadriceps muscle. Three measuring points per leg were marked with a permanent marker pen for scanning in the transverse and sagittal plane to make sure that the exact same measuring point was measured across time [31]: (1) lateral point: in the middle of the distance between the superior border of the greater trochanter and the lateral knee joint space; (2) ventral point: on a line from the anterior superior iliac spine to the superior border of the patellar base, on the same level as the lateral point; (3) medial point: on a line from the pubis to the medial knee joint space, on the same level as the lateral point. Frequency was set at 6 MHz. A pillow was placed under the patient’s knees to ensure muscular relaxation. A gel pad (Geliperm 260 × 120 × 3.3 mm, Geistlich Pharma, Wolhusen, Switzerland) placed on the patient’s thigh and additional ultrasound gel was used to apply the least amount of pressure necessary while scanning until the muscles were neatly displayed. Prior to trial start, inter-examiner variation in MLT scanning between the five trained assessors was assessed on 24 measuring points. The intra-class correlation coefficient (ICC) for the five assessors was 0.9 (95 % CI, 0.82 to 0.95) (*P* < 0.001). The five nonblinded assessors effectuated the ultrasound scans and muscle strength assessment and then applied the electrodes for NMES or sham stimulation. During the recruitment phase, none of the nonblinded assessors had access to any actual MLT values. Thus it appears unlikely that any observer bias was introduced by the nonblinded assessors who effectuated the ultrasound scans. After the end of data collection, MLT was evaluated with the software EchoPAC (General Electric Healthcare). The two evaluators were blinded to whether the patient belonged to the NMES or control group. Scans were evaluated per patient from the first to the last taken ultrasound scan to ensure consistency since muscular form is very individual. In that way, the level of the fascia lata could be discerned consistently in each patient. In a pilot testing we observed that the intramuscular fascia cannot be precisely delimitated from the muscular parts in patients during early ICU stay (Fig. 1 and Additional file 1). Thus we opted to measure the entire muscle layer thickness from the fascia lata (or deep fascia) to the femur, because these two landmarks can be precisely delineated. Beginning in the exact center of the scan at the level of the lowest layer of the fascia lata, MLT was measured down to the femur drawing the shortest possible line. At the lateral measuring point in the transverse and sagittal plane, MLT of vastus lateralis and vastus intermedius muscles was measured from the lowest layer of the fascia lata to the femur (Fig. 1 and Additional file 1). Similarly, MLT of rectus femoris and vastus intermedius muscles was measured in both planes at the ventral measuring point and MLT of vastus medialis and vastus intermedius muscles at the medial measuring point. MLT measurement in both the sagittal and transverse plane of one measuring point is shown in Additional file 1. MLT measured in the transverse plane highly correlated with the corresponding MLT measured in the sagittal plane (*r* = 0.97, *P* < 0.001). We averaged the tranverse and sagittal measurements for each measuring point. This accounts for interscan variation per measuring point. Since preliminary results indicated that the mean of all six measuring points in both planes is representative of all 12 measurements, mean MLT of all 12 measurements was calculated for each observation day.

#### Muscle strength

For all patients, muscle strength was measured on postoperative day 1 or as soon as they were awake, every day until ICU discharge and at hospital discharge. The patient was considered awake when he could follow orders, which is necessary for evaluation of active muscle contractions. Patients in cohort A had an additional assessment of muscle strength before surgery. Based on the diagnostic criteria of ICUAW, which is established when mean MRC score of all testable muscle groups is below 4 [5], muscle strength was evaluated in all muscle groups of the upper and lower extremities using the MRC scale [32]. Mean MRC score of all muscle groups was calculated [5]. In addition quadriceps mean MRC score was calculated as mean MRC score of knee extension and hip flexion of both legs to evaluate the function of the electrically stimulated quadriceps muscles. According to the MRC scale [33], mean MRC score ranges from a minimum of 0 to a maximum of 5 points. Bilateral grip strength was measured with a Jamar hand dynamometer (Jamar, Duluth, MN, USA). Patients were asked to perform the hand dynamometer test twice for each side and the highest value was noted for each side.

### Functional outcomes

For all patients, the Functional Independence Measure (FIM) score [34], the Timed Up and Go test [35] and the 12-Item Short Form (SF-12) health survey [36] were evaluated at hospital discharge. The average mobility level proposed by Brown [37] was assessed at ICU and hospital discharges. Patients in cohort A had an additional assessment of all functional outcomes before surgery.

### Patient satisfaction

At ICU discharge, patients were asked if the NMES/sham stimulation was comfortable or uncomfortable.

### Statistical analysis

In the previous Catastim 1 trial [24], MRC muscle strength was evaluated for hip flexion: mean changes in MRC score from the first postoperative day until ICU discharge were −0.80 points [standard deviation (SD), 0.70 points] in the NMES group (*n* = 20) versus −1.75 points (SD, 1.52 points) in the control group out of a maximum MRC score of 5 points (*n* = 20) (*P* = 0.015). Based on these results, sample size calculation with Stata yielded 25 patients per group at a two-sided significance level of α = 0.05 and 80 % power. Assuming a dropout rate of 20 %, 60 patients needed to be randomized.

All data was analyzed according to the intention-to-treat principle with no imputation for any missing data. Normality was assessed with Kolmogorov-Smirnov and Shapiro-Wilk statistics. If quantitative variables were normally distributed, they were expressed as mean ± SD or 95 % confidence interval (CI). If they were not normally distributed, median (range) was indicated.

In previous muscle ultrasound studies, linear regression analyses were performed [7, 27, 28]. Similarly, we presented results of actual raw data (Additional file 2 and Table 7) and results based on linear regression modeling (Tables 2, 3, 4, 5, 6 and Additional file 3). Linear mixed models were used to assess the treatment effects while accounting for the repeated measurements per patient. Thus, the random effect ‘patient’ was defined in each model. Linear mixed models were calculated for mean MLT, mean MRC score of all muscle groups, quadriceps mean MRC score and grip strength. Fixed effects of interest were postoperative day or study day, NMES/control group, interaction between NMES/control group and postoperative day, and daily fluid balance in the ICU. Fixed effects were either considered as quantitative or categorical variables: for example, the postoperative day or the daily fluid balance at the ICU were quantitative variables. The study day (preoperative day, first postoperative day, ICU discharge, hospital discharge) or the group (NMES group, control group) were categorical variables. By definition the results presented as linear mixed models are derived statistics by linear regression modeling (‘proc mixed’ in SAS software version 9.4; SAS Institute Inc., Cary, NC, USA).

Because of bandages to protect arterial catheters, patients were not always able to perform every movement in all 12 muscle groups. If there were missing values for muscle groups, mean MRC score was calculated for the muscle groups that could be assessed. Similarly, due to armboards because of radial arterial catheter, patients were not always able to perform the hand dynamometer test on both sides. Thus, either the right or left grip strength was chosen for statistical analysis per patient according to the lowest number of missing values over all study days on each side. If the number of missing values was equal for right and left grip strength for a patient, either the right or left grip strength was chosen according to the highest mean grip strength over all study days on each side.

In addition, intraoperative fluid balance, changes over time in MLT or mean MRC score were compared between both groups with independent-groups *t* tests or Mann-Whitney *U* tests as appropriate. Change in MLT was correlated with the cumulative fluid balance in the first three postoperative days with Pearson product-moment correlation.

The secondary functional outcomes were evaluated in a sensitivity analysis for patients seen both on preoperative day and at hospital discharge (*n* = 12) in order to evaluate whether preoperative functional levels could be regained at hospital discharge. Changes over time in secondary functional outcomes were analyzed with Wilcoxon signed-rank tests. Changes in functional outcomes were compared between both groups with Mann-Whitney *U* tests. The patient satisfaction related to the intervention was evaluated in 42 patients with Pearson’s chi-squared test or Fisher’s exact test as appropriate.

Significance level was set at 0.05. All *P* values were two-tailed. For statistical analysis, SPSS version 22 (IBM Corp., Armonk, NY, USA) and SAS version 9.4 were used. For figure construction, GraphPad Prism version 6.0 (GraphPad Software, Inc., La Jolla, CA, USA) was used.

## Results

### Patients

**Fig. 2:**
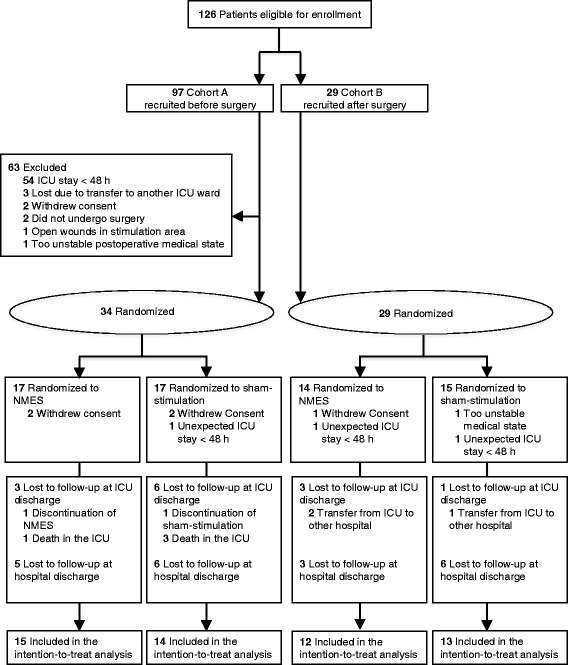
Flow diagram of the Catastim 2 trial

Fifty-four patients were included in the intention-to-treat analysis: 27 belonged to the NMES group and 27 to the control group (Fig. 2). All baseline characteristics were comparable in both groups except for dobutamine and noradrenaline doses in the ICU (Table 1).

**Table 1:** Baseline characteristics of patients

	NMES group (*n* = 27)	Control group (*n* = 27)
Age, mean (SD), y	63.3 (15.5)	69.7 (13.1)
Male sex, no. (%)	18	20
Body mass index, mean (SD)	27.6 (3.7)	27.7 (4.6)
SAPS II at postoperative day 1, median (range)	26.0 (7–46)	24.0 (7–47)
SOFA at postoperative day 1, median (range)	9.0 (1–15)	7.0 (1–11)
Period ventilated, median (range), d^*a*^	2 (1–7)	2 (1–15)
ICU length of stay, median (range), d^*b*^	6 (3–23)^*c*^	7 (3–213)^*c*^
ICU readmission, no. (%)	4 (14.8)	3 (11.1)
ICU readmission, median (range), d^*b*^	2 (1–35)	6 (1–41)
Hospital length of stay, median (range), d^*b*^	22 (4–84)^*d*^	19 (9–213)^*d*^
Mortality, no (%)		
in the ICU	1 (3.7)	3 (11.1)
at the surgical ward	0	0
Single surgical procedure, no. (%)^*e*^	17 (63.0)	17 (63.0)
Double surgical procedure, no. (%)^*e*^	7 (25.9)	7 (25.9)
Triple surgical procedure, no. (%)^*e*^	3 (11.1)	3 (11.1)
Surgery type, no. of procedures (% in relation to total no. of procedures)		
Aortic valve replacement	9 (22.5)	11 (27.5)
Coronary artery bypass grafting	11 (27.5)	8 (20)
Heart transplantation	6 (15)	5 (12.5)
Other cardiothoracic surgery type^*f*^	4 (10)	4 (10)
Mitral valve replacement	2 (5)	5 (12.5)
Mitral valve reconstruction	4 (10)	3 (7.5)
Tricuspid valve reconstruction	3 (7.5)	2 (5)
Bentall procedure	1 (2.5)	2 (5)
Comorbidities present in 96.3 % of the study population, no. (%)		
Coronary heart disease	12 (44.4)	8 (29.6)
Hypertension	9 (33.3)	7 (25.9)
Myocardial infarction	7 (25.9)	4 (14.8)
Atrial fibrillation	2 (7.4)	8 (29.6)
Diabetes	5 (18.5)	4 (14.8)
Obesity	3 (11.1)	6 (22.2)
Chronic obstructive pulmonary disease	6 (22.2)	3 (11.1)
Hyperlipidemia	4 (14.8)	4 (14.8)
Malignoma	2 (7.4)	5 (18.5)
Carotid artery stenosis	2 (7.4)	2 (7.4)
Patients receiving medication in the ICU, no. (%)		
Opioids	26 (96.3)	26 (96.3)
Insulin	25 (92.6)	26 (96.3)
Dobutamine	21 (77.8)	17 (63)
Noradrenaline	18 (66.7)	18 (66.7)
Glucocorticoids	11 (40.7)	6 (22.2)
Benzodiazepines	6 (22.2)	6 (22.2)
Neuromuscular blockers	4 (14.8)	2 (7.4)
Intake days, median (range), d		
Opioids	3 (1–10)	3 (1–14)
Insulin	4 (1–9)	4 (1–14)
Dobutamine	4 (2–12)	5 (1–14)
Noradrenaline	2 (1–9)	4.5 (1–14)
Glucocorticoids	3 (1–7)	3.5 (1–14)
Benzodiazepines	1 (1–4)	1 (1–9)
Neuromuscular blockers	1 (1–1)	1 (1–1)
Dose, median (range), μg/kg/min		
Dobutamine	2.78 (0.52–7.59)^*g*^	4.12 (0.82–10.53)^*g*^
Noradrenaline	0.07 (0.01–0.54)^*h*^	0.12 (0.002–0.88)^*h*^

*NMES* neuromuscular electrical stimulation, *SD* standard deviation, *SAPS II* Simplified Acute Physiology Score II, *SOFA* Sequential Organ Failure Assessment, *ICU* intensive care unit

^*a*^The ventilation period was calculated as the number of calendar days from the day of surgery (counted as 1 day) to the last day of ventilation (also counted as 1 day). All patients were ventilated when transferred from the operating room to the ICU

^*b*^The time period was calculated as the number of calendar days from the day of ICU/hospital (re)admission (counted as 1 day) to the day of ICU/hospital discharge (also counted as 1 day unless discharged on the same day as the (re)admission to ICU)

^*c*^ *P* = 0.46

^d^ *P* = 0.60

^*e*^Example of single surgical procedure: aortic valve replacement; example of double surgical procedure: aortic valve replacement + coronary artery bypass grafting; example of triple surgical procedure: aortic valve replacement + mitral valve reconstruction + coronary artery bypass grafting

^*f*^Atrial septal defect II closure, pericardial tamponade, lung transplantation, resection of cardiac aneurysm, aortic arch replacement, Glenn procedure, Ross procedure, replacement of ascending aorta

^*g*^ *P* < 0.001

^h^ *P* = 0.01

### NMES

Median duration of the electrical or sham stimulation was 4 days (range, 2–13 days) in the NMES group and 4 days (range, 1–14 days) in the control group. In the NMES group, median NMES intensities were 40.5 mA (range, 2–100 mA) for the right thigh and 40 mA (range, 5–120 mA) for the left thigh. In terms of adherence, only 9 out of 145 NMES sessions and 6 out of 154 sham stimulation sessions could not have been carried out.

### Muscle layer thickness (MLT)

MLT was evaluated in 53 out of 54 patients. Due to technical reasons, ultrasound scans of one patient could not be saved. MLT was not evaluated in 210 of all 2825 ultrasound scans, because the muscles of the thigh could not be clearly delimited (Additional file 4). MLT increased from preoperative day to the first postoperative day by 0.41 cm (95 % CI of estimate, 0.24 to 0.59 cm) (*P* < 0.001) (Table 3). Intraoperative fluid balance was 4856.5 ± 2637.4 ml (mean ± SD) in the NMES group (*n* = 26) and 4111.9 ± 1766.6 ml (mean ± SD) in the control group (*n* = 26) (*P* = 0.24). During the first three postoperative days, there was a positive correlation between change in MLT and cumulative fluid balance (*r* = 0.43, *P* = 0.01) (*n* = 41). From the first postoperative day, MLT decreased by 0.08 cm (95 % CI of estimate, −0.11 to −0.06 cm) per day (*P* < 0.001). The change in MLT of −0.08 cm per day is the result of the linear regression from the first postoperative day where each patient is considered as a random factor. Overall this means that MLT decreases by 8 mm (95 % CI, −11 to −6 mm) in 10 days by linear regression modeling (Table 2). This corresponds to a 25 % decrease in muscle layer thickness in 10 days. The NMES intervention had no significant effect on MLT (Table 2). At ICU discharge, MLT was about 0.18 cm (95 % CI of estimate, −0.004 to 0.37 cm) higher than on preoperative day (*P* = 0.055) (Table 3). At hospital discharge, MLT was about 0.28 cm (95 % CI of estimate, −0.49 to −0.06 cm) lower than on preoperative day (*P* = 0.01) (Table 3). Time variation of actual MLT values in individual patients is shown in Additional file 2.

**Table 2:** Linear mixed model for MLT from the first postoperative day for a maximum of 14 postoperative days (53 patients, 183 observations)

	Estimate of MLT in cm (95 % CI)	*P* value
Intercept	3.87 (3.58 to 4.15)	<0.001
Postoperative day	−0.08 (−0.11 to −0.06)	<0.001
Control group	Reference	.
NMES group	−0.18 (−0.59 to 0.23)	0.38
Postoperative day × Control group	Reference	.
Postoperative day × NMES group	0.02 (−0.01 to 0.06)	0.21
Daily fluid balance in the ICU	−0.04 (−0.08 to 0.01)	0.09

*MLT* muscle layer thickness, *CI* confidence interval, *NMES* neuromuscular electrical stimulation, *ICU* intensive care unit

**Table 3:** Linear mixed model for MLT on four important study days (53 patients, 141 observations)

	Estimate of MLT in cm (95 % CI)	*P* value
Intercept	3.26 (2.99 to 3.52)	<0.001
Preoperative day	Reference	.
First postoperative day	0.41 (0.24 to 0.59)	<0.001
ICU discharge	0.18 (−0.004 to 0.37)	0.055
Hospital discharge	−0.28 (−0.49 to −0.06)	0.01
Control group	Reference	.
NMES group	−0.16 (−0.49 to 0.16)	0.32

*MLT* muscle layer thickness, *CI* confidence interval, *ICU* intensive care unit, *NMES* neuromuscular electrical stimulation

### Muscle strength

Out of all 54 patients, 36 patients were awake on postoperative day 1, 7 patients on postoperative day 2 or 3, 8 patients on postoperative days 4 to 7 in order to perform the first postoperative MRC measurement in the ICU. Three patients remained sedated during the first 14 postoperative days and were not able to undergo MRC evaluation during their ICU stay. All patients in cohort A, who were assessed before surgery, had normal muscle strength (mean MRC score of 4.92 points (3.83 to 5 points), median (range), in the NMES group versus 4.92 points (3.67 to 5 points), median (range), in the control group) (*P* = 0.96). Mean MRC score of all muscle groups decreased from preoperative day to the first postoperative day by 0.57 points (95 % CI of estimate, −0.78 to −0.36 points) (*P* < 0.001) (Table 5). The decrease in mean MRC score of all muscle groups from preoperative day to postoperative day 1 was significantly higher in the NMES group [median (range): −0.65 points (−1.82 to 0 points)] than in the control group [median (range): −0.11 points (−2.36 to 0.18 points)] (*P* = 0.046). Thus, even before the NMES intervention began, patients randomized to the NMES group had a weaker mean MRC of −0.45 points (95 % CI of estimate, −0.88 to −0.03 points, *P* = 0.04) than patients randomized to the control group at the starting point, which was the first postoperative day (Table 4). This is unrelated to the NMES intervention because the intervention started only afterwards. After the onset of the NMES intervention, patients in the NMES group had a 4.5 times higher slope in recovering muscle strength than patients in the control group by linear regression modeling [0.09 MRC points per day (95 % CI of estimate, 0.03 to 0.14 MRC points per day) in the NMES group versus 0.02 MRC points per day (95 % CI of estimate, −0.02 to 0.05 MRC points per day) in the control group] (Table 4). Finally, mean MRC score of all muscle groups regained preoperative values at hospital discharge independent of group allocation (*P* = 0.43) (Table 5). Time variation of actual mean MRC score of all muscle groups in individual patients is shown in Additional file 2. Analysis of quadriceps mean MRC showed similar results to those of mean MRC of all muscle groups (Additional file 3). Patients had lost 4 kilogram-force (95 % CI of estimate, −6.90 to −1.09 kilogram-force) in hand grip strength at hospital discharge compared to preoperative day (*P* = 0.008) (Table 6). The NMES intervention had no significant effect on grip strength (Table 6).

**Table 4:** Linear mixed model for mean MRC of all muscle groups^a^ from the first postoperative day for a maximum of 14 postoperative days (51 patients, 220 observations)

	Estimate of mean MRC in points^a^ (95 % CI)	*P* value
Intercept	4.10 (3.80 to 4.39)	<0.001
Postoperative day	0.02 (−0.02 to 0.05)	0.40
Control group	Reference	.
NMES group	−0.45 (−0.88 to −0.03)	0.04
Postoperative day × Control group	Reference	.
Postoperative day × NMES group	0.09 (0.03 to 0.14)	0.002

Days of ICU and hospital discharge, where no NMES was applied anymore, were excluded in this model

The linear mixed model for mean MRC in Table 4 reads as follows:

MRC = 4.10 + (0.02 × postoperative day) - (0.45 × NMES group) + (0.09 × postoperative day × NMES group)

0.02 is the slope of MRC time variation in the control group, which is the reference group: for each postoperative day, MRC increases by 0.02 points (95 % CI, −0.02 to 0.05 points) in the control group (*P* = 0.40)

0.45 represents the lower starting point in the NMES group on the first postoperative day before the NMES intervention began: on the first postoperative day, MRC was about −0.45 points (95 % CI, −0.88 to −0.03 points) lower in the NMES group than in the control group (*P* = 0.04)

0.09 is the slope of MRC time variation in the NMES group: the slope of MRC time variation is 4.5 times higher than the slope in the control group (*P* = 0.002)

*MRC* Medical Research Council, *CI* confidence interval, *NMES* neuromuscular electrical stimulation

^a^According to the MRC scale [33], mean MRC score ranges from a minimum of 0 to a maximum of 5 points

**Table 5:** Linear mixed model for mean MRC of all muscle groups on four important study days (51 patients, 130 observations)

	Estimate of mean MRC in points^a^ (95 % CI)	*P* value
Intercept	4.69 (4.45 to 4.93)	<0.001
Preoperative day	Reference	.
First postoperative day	−0.57 (−0.78 to −0.36)	<0.001
ICU discharge	−0.27 (−0.48 to −0.06)	0.01
Hospital discharge	0.10 (−0.15 to 0.35)	0.43
Control group	Reference	.
NMES group	0.01 (−0.26 to 0.28)	0.92

The linear mixed model for mean MRC in Table 5 reads as follows:

On the first postoperative day mean MRC was about −0.57 points (95 % CI, −0.78 to −0.36 points) lower than on preoperative day, which is the reference day (*P* < 0.001)

At ICU discharge mean MRC was about −0.27 points (95 % CI, (−0.48 to −0.06 points) lower than on preoperative day (*P* = 0.01)

At hospital discharge mean MRC was not different from mean MRC on preoperative day (*P* = 0.43)

On preoperative day, at the first postoperative day, at ICU discharge and at hospital discharge there were no differences in mean MRC between the NMES and control group (*P* = 0.92)

*MRC* Medical Research Council, *CI* confidence interval, *ICU* intensive care unit, *NMES* neuromuscular electrical stimulation

^a^According to the MRC scale [33], mean MRC score ranges from a minimum of 0 to a maximum of 5 points

**Table 6:** Linear mixed model for grip strength measured by hand dynamometer on four important study days (49 patients, 127 observations)

	Estimate of mean MRC in kilogram-force (kgf) (95 % CI)	*P* value
Intercept	30.18 (25.63 to 34.73)	<0.001
Preoperative day	Reference	.
First postoperative day	−11.55 (−13.98 to −9.11)	<0.001
ICU discharge	−8.00 (−10.47 to −5.52)	<0.001
Hospital discharge	−4.00 (−6.90 to −1.09)	0.008
Control group	Reference	.
NMES group	0.89 (−5.16 to 6.94)	0.77

*MRC* Medical Research Council, *CI* confidence interval, *ICU* intensive care unit, *NMES* neuromuscular electrical stimulation

### Functional outcomes

The secondary functional outcomes were evaluated for patients seen both on preoperative day and at hospital discharge (*n* = 12). The average mobility level and FIM score significantly decreased from preoperative day to hospital discharge (Table 7). On the other hand, the Timed Up and Go Test as well as the mental component score (MCS-12) and physical component score (PCS-12) of the SF-12 regained preoperative levels at hospital discharge (Table 7). Changes in all functional outcomes from preoperative day to ICU or hospital discharge were not significantly different between both groups.

**Table 7:** Functional outcomes on four important study days (12 patients)

	Preoperative day	ICU discharge	Hospital discharge	*P* for change from preoperative day to hospital discharge
Average mobility level, median (range)	12 (12–12)	2 (0–11)^a^	12 (8–12)^b^	0.04
FIM score, median (range)	125.0 (119–126)		122.0 (82–126)	0.02
Timed Up and Go Test, median (range)	11.5 (7–20)		11.5 (1–28)	0.24
MCS-12, median (range)	52.82 (25.05–64.63)		51.91 (29.42–64.08)	0.88
PCS-12, median (range)	40.02 (17.91–61.61)		32.58 (27.46–45.90)	0.53

*ICU* intensive care unit, *FIM F*unctional Independence Measure, *MCS-12* mental component score of the SF-12, *PCS-12* physical component score of the SF-12

^a^ *P* for change from preoperative day to ICU discharge: 0.002

^b^ *P* for change from ICU discharge to hospital discharge: 0.002

### Patient satisfaction

Forty-two patients were asked about their satisfaction with the NMES/sham stimulation. Out of 42 patients, 12 patients in the NMES group and 5 patients in the control group had a comfortable sensation (*P* = 0.03). Five patients in the NMES group and no patient in the control group had a feeling of discomfort (*P* = 0.048).

## Discussion

In this randomized controlled trial, NMES had no effect on MLT, but was associated with a higher rate in regaining muscle strength during the ICU stay. At hospital discharge, all patients regained preoperative levels of muscle strength evaluated with the MRC scale, but still showed residual functional disability and decreased MLT compared to pre-ICU levels.

### Muscle layer thickness (MLT)

The increase in MLT from preoperative day to postoperative day 1 can be attributed to the surgically induced inflammation and to the positive intraoperative fluid balance. Fluid is entering the muscle, since cumulative fluid balance and change in MLT positively correlate in the first three postoperative days. Postoperatively, MLT progressively decreased until ICU discharge, but was still higher at ICU discharge than on preoperative day. This implies that the intramuscular edema regressed, but was still present at ICU discharge. Only at hospital discharge, MLT was lower than on preoperative day. This clearly indicates that also MLT was lost. It is not possible to tell at what time muscle loss started, since measurement of MLT was highly affected by intramuscular edema.

In the Catastim 2 study, NMES had no effect on MLT. Edema could have led to dissipation of the electrical current so that the current reaching the intramuscular motor nerve branches was not sufficient to generate muscle contractions. Segers [38] demonstrated that patients with high edema had insufficient muscle contractions during NMES. Segers found that patients had more sufficient NMES muscle contractions at the beginning of their ICU stay than after 1 week of ICU stay. However they did not analyze whether this was attributed to changes in development of edema or sepsis. In our study the positive correlation between cumulative fluid balance and change in MLT during the first three postoperative days suggests that edema is predominant in the early stage of surgical critical illness. Hence the effects of NMES may be rather reduced in the first days of critical illness. In the first ICU week, up to 62 % of all patients had a nonexcitable muscle membrane [39], which may also attenuate the effects of NMES.

So far, only three studies [9, 19, 40] showed an effect of NMES on muscle mass in critically ill patients. However variation in muscle mass was not compared to pre-ICU values and fluid status was not considered as a confounder for measurement of muscle mass in these studies [9, 19, 40]. The Catastim 2 study indicates that the postoperative decrease in MLT at the ICU is not only due to loss of muscle mass, but also to reduction of intramuscular edema.

### Muscle strength

The decrease in MRC strength from preoperative day to postoperative day 1 is most likely due to the surgical event and the postoperative sedation. Even before the NMES intervention began, the decrease in MRC from preoperative day to postoperative day 1 was coincidentally more pronounced in patients randomized to the NMES intervention than in patients randomized to the control group. After the onset of the NMES intervention, patients in the NMES group had a 4.5 times higher slope in recovering muscle strength during the ICU stay than patients in the control group. This indicates that the NMES intervention promotes a faster regain of muscle strength during the ICU stay. Still, both groups were able to regain preoperative MRC values at hospital discharge. Nevertheless, a faster recovery may be beneficial and is an interesting result. Moreover, it shows that it is important to consider the factor time in the analysis of time variation of muscle strength in the ICU. No previous study [16–18, 21, 22] yet accounted for an interaction between NMES and observation day in a linear regression model.

Similar to our results, Kho’s study [16] showed a higher increase in MRC from ICU awakening to ICU and hospital discharges in the NMES group. In Kho’s study, the absolute MRC values at hospital discharge were not different between both groups. Unfortunately, Kho did not assess MRC prior to the ICU in order to determine whether both groups could regain their preoperative muscle strength levels. Other studies also showed positive effects of NMES on the MRC score in ICU patients [16–18, 20–22]. In line with these previous studies, the Catastim 2 study shows that NMES contributes to a faster recovery of muscle strength during the ICU stay.

### Functional outcomes

In contrast to the recovery of muscle strength, patients did not regain their preoperative levels of mobility (average mobility level) and functional abilities (FIM score) at hospital discharge, independent of group allocation. This indicates that muscle strength and mobility may not be similarly affected by ICUAW. Similar to our results, Kho et al. [16] showed that the preoperative functional status score for the intensive care (FSS-ICU) score, which is similar to the FIM score, was not regained at hospital discharge, independent of group assignment. These functional disabilities at hospital discharge still persist 2 months to 5 years later as other trials have shown. After hospital discharge patients may regain their muscle strength, but not their functional abilities: patients with lower muscle strength at hospital discharge regained strength within 12 months [41]. However patients with lower muscle strength at hospital discharge still had lower SF-36 physical component score and lower 6-minute walking distance (6MWD) 3, 6, 12 and 24 months after ICU discharge [41]. Similarly, ICU survivors spent only 3 % of their time walking and 90 % of their time inactive at 2 months after ICU discharge [42]. Their 6MWD was also decreased [42]. Likewise, Wieske [43] showed that patients with lower muscle strength during their ICU stay had lower SF-36 physical functioning 6 months after ICU discharge. Needham [44] also showed that acute lung injury survivors had lower 6MWD and SF-36 at 6 and 12 months after hospital discharge in comparison to normal predicted values. Even at 5 years after ICU discharge, 6MWD and SF-36 were reduced [3]. These impressive results clearly show that ICUAW is a public health issue going beyond hospital discharge, which calls for prophylactic or therapeutic measures. In our study, NMES had no effect on functional outcomes at hospital discharge. Yet, our sensitivity analysis for the secondary functional outcomes only included a small patient subcategory (*n* = 12) seen both on preoperative day and at hospital discharge. The sensitivity analysis was chosen in order to determine whether preoperative functional levels could be regained at hospital discharge. Positive effects of early mobilization on functional outcomes were shown in other studies: Schweickert [15] showed that sedation interruptions and physical therapy led to better functional outcomes at hospital discharge. Abdellaoui [18] found an increase in 6MWD in the NMES group. Zanotti [22] observed that NMES reduced the number of days needed to transfer from bed to chair. Parry [45] showed that combined electrical stimulation and cycling in critically ill patients led to a shorter delirium period.

### Limitations

Even before the NMES intervention started, patients randomized to the NMES group had a higher decrease in MRC score than patients randomized to the control group. This higher decrease in MRC at the first postoperative day may also contribute to a higher rate in recovering MRC in the NMES group during the ICU stay. Hence, the higher rate in recovering MRC during the ICU stay may not be due to the NMES intervention alone, but also partly to the coincidentally higher decrease in MRC from preoperative day to the first postoperative day in the NMES group.

The average mobility level was initially suggested by Brown in 2004 in the *Journal of the American Geriatrics Society* [37]. The average mobility level proposed by Brown is not mentioned among measurement instruments for impairments in the critically ill patient in Parry’s systematic review [46]. There may be better functional scores to assess mobility. On the other hand, the FIM score is recommended in Parry’s systematic review [46].

### Outlook

Visualizing or palpating the NMES contractions was hardly possible in patients with high fluid retention in our study and in Seger’s study [38]. Therefore it should be verified by ultrasonography whether muscles are contracting sufficiently during NMES in the presence of edema.

The assessment of baseline values prior to surgery or acute illness is of paramount importance in order to adjust for confounders related to surgery such as inflammation, fluid balance and sedation. Fluid balance should be taken into account when MLT is assessed by ultrasonography. Due to the lack of combined assessment of preoperative muscle mass values and fluid balance in previous studies, the formation and regression of intramuscular edema have never been shown before and need to be thoroughly addressed in future.

In line with previous studies [41], it is not the loss in muscle strength or mass but primarily the loss in functional outcomes which may play the more significant and patient-centered public health issue. Future studies should especially study patient-centered functional outcomes such as FIM, mobility and health-related quality of life (HRQoL).

## Conclusions

NMES had no effect on MLT, but was associated with a higher rate in regaining muscle strength during the ICU stay. Regression of intramuscular edema during the ICU stay interfered with measurement of changes in MLT. At hospital discharge, all patients had regained preoperative levels of muscle strength evaluated with the MRC scale, but still showed residual functional disability and decreased MLT compared to pre-ICU levels.

## Key messages

- The NMES intervention had no effect on muscle layer thickness, but was associated with a higher rate in regaining muscle strength during the ICU stay.
- Patients regained preoperative levels of muscle strength evaluated with the MRC scale by hospital discharge, but still had residual functional disability. This was independent of group allocation.
- The postoperative decrease in muscle layer thickness is not only due to loss of muscle mass, but also to reduction of intramuscular edema.

## Additional files

Additional file 1: **Ultrasound scan of the left thigh at the lateral measuring point in the transverse and sagittal plane (Patient no. 104, control group) on postoperative day 3.** (TIF 798 kb)

Additional file 2: **Time variation of MLT and mean MRC of all muscle groups in individual patients.** Days of ICU and hospital discharge are included. (PDF 837 kb)

Additional file 3: **Linear mixed models for quadriceps mean MRC** ^**a**^ **Table S1A.** Linear mixed model for quadriceps mean MRC from the first postoperative day for a maximum of 14 postoperative days (51 patients, 219 observations) Days of ICU and hospital discharge, where no NMES was applied anymore, were excluded in this model. **Table S1B.** Linear mixed model for quadriceps mean MRC on four important study days (51 patients, 130 observations). (PDF 89.3 kb)

Additional file 4: **Ultrasound scan of the left thigh at the medial measuring point in the transverse plane (Patient no. 84, control group).** MLT was not evaluated on postoperative day 1. (TIF 1045 kb)

## Abbreviations

6MWD: 6-Minute walking distance
CI: Confidence interval
FIM: Functional Independence Measure
FSS-ICU: Functional status score for the intensive care unit
HRQoL: Health-related quality of life
ICC: Intra-class correlation coefficient
ICUAW: Intensive care unit-acquired weakness
MCS-12: Mental component score of the SF-12
MLT: Muscle layer thickness
MRC: Medical Research Council
NMES: Neuromuscular electrical stimulation
PCS-12: Physical component score of the SF-12
SAPS II: Simplified Acute Physiology Score II
SD: Standard deviation
SF-12: 12-Item Short Form health survey
SF-36: 36-Item Short Form health survey
