# Supplementary figures and images for: Muscle mass, strength and functional outcomes in critically ill patients after cardiothoracic surgery: does neuromuscular electrical stimulation help? The Catastim 2 randomized controlled trial
Source: Crit Care. 2016 Jan 29;20:30. doi: 10.1186/s13054-016-1199-3 (PMC4733279; doi:10.1186/s13054-016-1199-3)

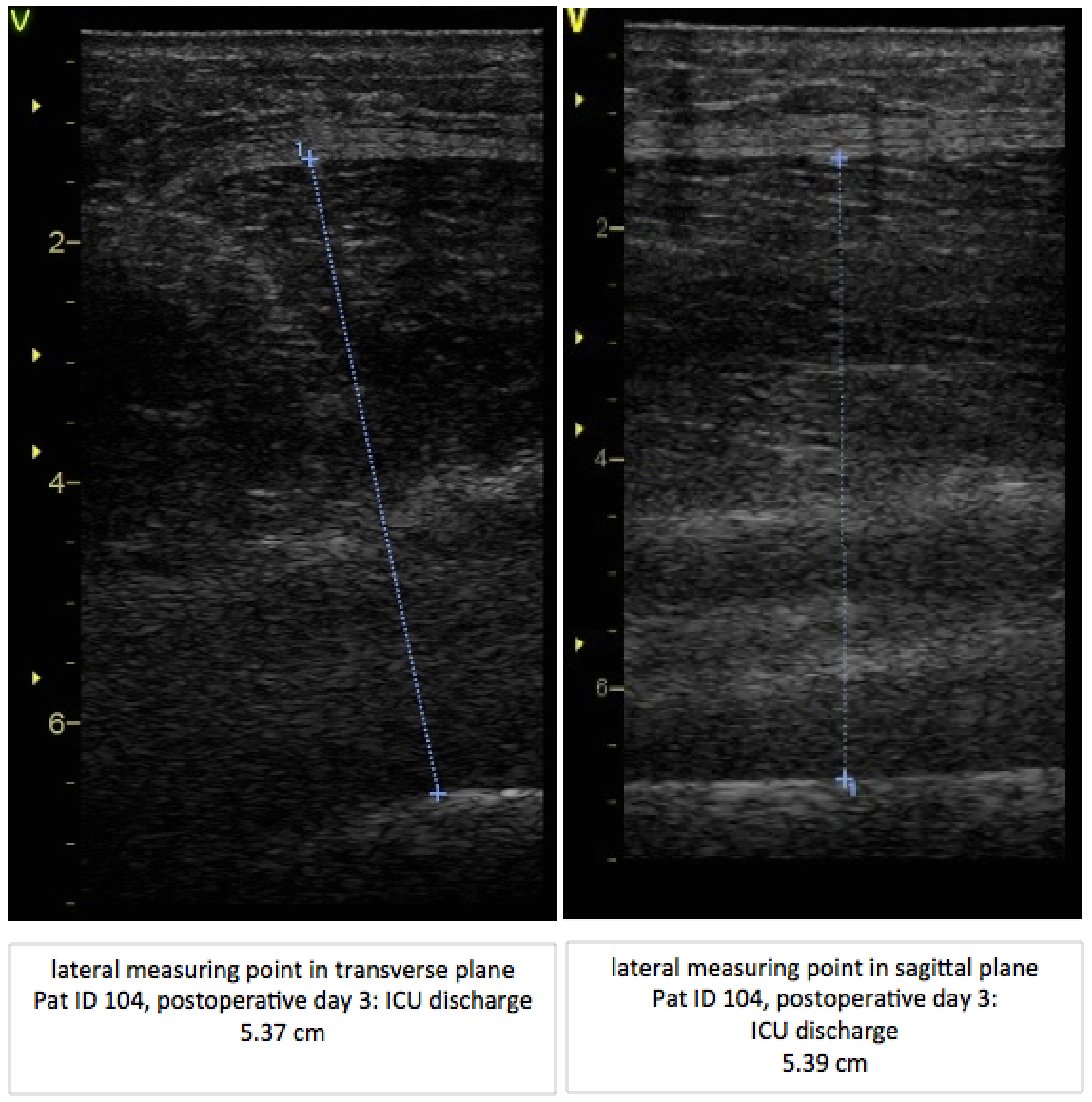

Supplement: Supplementary file 1 — Ultrasound scan of the left thigh at the lateral measuring point in the transverse and sagittal plane (Patient no. 104, control group) on postoperative day 3. (TIF 798 kb) [file 13054_2016_1199_MOESM1_ESM.tif]

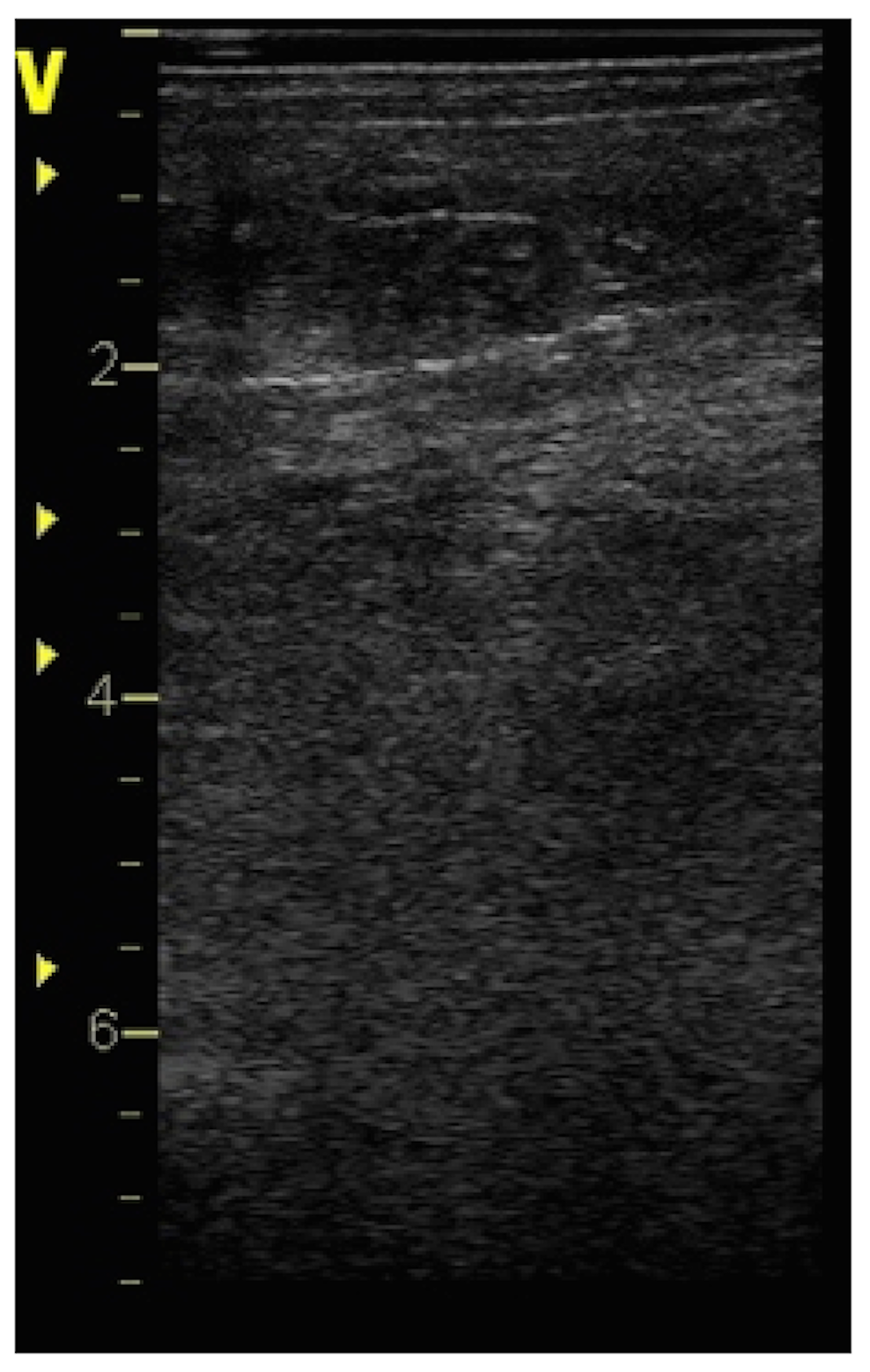

Supplement: Supplementary file 4 — Ultrasound scan of the left thigh at the medial measuring point in the transverse plane (Patient no. 84, control group). MLT was not evaluated on postoperative day 1. (TIF 1045 kb) [file 13054_2016_1199_MOESM4_ESM.tif]
