# Supplementary material for: Muscle mass, strength and functional outcomes in critically ill patients after cardiothoracic surgery: does neuromuscular electrical stimulation help? The Catastim 2 randomized controlled trial
Source: Crit Care. 2016 Jan 29;20:30. doi: 10.1186/s13054-016-1199-3 (PMC4733279; doi:10.1186/s13054-016-1199-3)
Supplement: Supplementary file 3 — Linear mixed models for quadriceps mean MRC a Table S1A. Linear mixed model for quadriceps mean MRC from the first postoperative day for a maximum of 14 postoperative days (51 patients, 219 observations) Days of ICU and hospital discharge, where no NMES was applied anymore, were excluded in this model. Table S1B. Linear mixed model for quadriceps mean MRC on four important study days (51 patients, 130 observations). (PDF 89.3 kb) [file 13054_2016_1199_MOESM3_ESM.pdf]

Additional File 3. Linear mixed models for quadriceps mean MRC[a]

Table S1A. Linear mixed model for quadriceps mean MRC from the first postoperative day for a maximum of 14 postoperative days (51 patients, 219 observations)
Days of ICU and hospital discharge, where no NMES was applied anymore, were excluded in this model.

	Estimate of quadriceps mean MRC in points[a] (95% CI)	*P* value
Intercept	4.22 (3.90 to 4.54)	< .001
Postoperative day	-0.01 (-0.06 to 0.04)	.63
Control group	reference	.
NMES group	-0.62 (-1.08 to -0.16)	.009
Postoperative day × Control group	reference	.
Postoperative day × NMES group	0.11 (0.05 to 0.18)	< .001

Table S1B. Linear mixed model for quadriceps mean MRC on four important study days (51 patients, 130 observations)

	Estimate of quadriceps mean MRC in points[a] (95% CI)	*P* value
Intercept	4.75 (4.50 to 5.01)	< .001
Preoperative day	reference	.
First postoperative day	-0.60 (-0.84 to -0.36)	< .001
ICU discharge	-0.27 (-0.50 to -0.03)	.03
Hospital discharge	0.12 (-0.16 to 0.39)	.39
Control group	reference	.
NMES group	-0.04 (-0.32 to 0.25)	.80

[a] Quadriceps mean MRC was calculated as mean of hip flexion and knee extension of both sides. According to the MRC scale [29], mean MRC score ranges from a minimum of 0 to a maximum of 5 points.
